# Quercetin prevents hepatic fibrosis by inhibiting hepatic stellate cell activation and reducing autophagy via the TGF-β1/Smads and PI3K/Akt pathways

**DOI:** 10.1038/s41598-017-09673-5

**Published:** 2017-08-24

**Authors:** Liwei Wu, Qinghui Zhang, Wenhui Mo, Jiao Feng, Sainan Li, Jingjing Li, Tong Liu, Shizan Xu, Wenwen Wang, Xiya Lu, Qiang Yu, Kan Chen, Yujing Xia, Jie Lu, Ling Xu, Yingqun Zhou, Xiaoming Fan, Chuanyong Guo

**Affiliations:** 1 Department of Gastroenterology, Shanghai Tenth People’s Hospital, Tongji University School of Medicine, Shanghai, 200072 China; 2 Department of Clinical Laboratory, Kunshan First People’s Hospital Affiliated to Jiangsu University, 215300 Kunshan, JiangSu China; 3 Department of Gastroenterology, Minhang Hospital, Fudan University, Shanghai, 201100 China; 4 Department of Gastroenterology, Shanghai Tenth Hospital, School of Clinical Medicine of Nanjing Medical University, Shanghai, 200072 China; 5 Department of Gastroenterology, Shanghai Tongren Hospital, Shanghai Jiao Tong University School of Medicine, Shanghai, 200336 China; 6 Department of Gastroenterology, Jinshan Hospital of Fudan University, Jinshan, Shanghai 201508 China

## Abstract

The aim of this study was to investigate the effect of quercetin on hepatic fibrosis, a characteristic response to acute or chronic liver injury. Mice were randomized to bile duct ligation (BDL) or carbon tetrachloride (CCl_4_) cirrhosis models. Quercetin (100 mg/kg or 200 mg/kg daily) was administered by gavage for 2 or 4 weeks. Liver tissue and blood samples were collected for histological and molecular analysis. The results of our experiments showed that quercetin reduced BDL or CCl_4_ liver fibrosis, inhibited extracellular matrix formation, and regulated matrix metallopeptidase (MMP)-9 and tissue inhibitor of metalloproteinase (TIMP)-1. Quercetin attenuated liver damage by suppressing the TGF-β1/Smads signaling pathway and activating the PI3K/Akt signaling pathway to inhibit autophagy in BDL- or CCl_4_- induced liver fibrosis. Quercetin prevented hepatic fibrosis by attenuating hepatic stellate cell activation and reducing autophagy through regulating crosstalk between the TGF-β1/Smads and PI3K/Akt pathways.

## Introduction

Hepatic fibrosis is characteristic of liver acute or chronic injury in response to diverse metabolic, viral, and toxic stimuli. Fibrosis ultimately results in cirrhosis and complications such as portal hypertension, liver failure,hepatocellular carcinoma^1^. Excessive deposition of extracellular matrix (ECM) proteins, including hyaluronic acid (HA), laminin (LN), and collagen occur during fibrogenesis along with activation of hepatic stellate cells (HSCs)^2^. HSCs are silent in normal liver tissue but are activated by hepatic injury, and change into myofibroblast-like cells during the fibrotic process. Activated HSCs secrete transforming growth factor (TGF)-β1, which induces collagen production that leads ECM accumulation, and they also up-regulate tissue inhibitors of metalloproteinases (TIMPs). Inhibition of matrix metalloproteinases (MMPs) results in reduced ECM degradation^3^. Controlling the activation of HSCs that occurs during hepatic fibrosis may have a therapeutic benefit^4–7^.

TGF-β1 is a pleiotropic cytokine involved in ECM production, immune response, embryogenesis, and cell-cycle control. The TGF-β1 signaling pathway is mediated by the SMAD family, but other effectors such as phosphatidylinositol-3 kinase (PI3K), mitogen-activated protein kinase (MAPK), and nuclear factor kB (NF-kB), which also have key roles in cell proliferation, apoptosis, differentiation and ECM synthesis may be involved, and can independently regulate SMAD expression^8^.

Autophagy, which has both death-promoting and survive-promoting function, is an important part in liver diseases^9–11^. There are many proteins associated with autophagy, such as Beclin-1,LC3 and P62. In the autophagy action, damaged organelles are encircled by membrane to form autophagosomes. And Beclin-1 combines with the diaphragm and generate the production of an Atgs complex. And then, LC3-|| binds to the diaphragm, promoting autophagosome membrane extension. Mature autophagosome finally form.

Quercetin (3,3,4,5,7-pentahydroxyflavone,QE), is a flavonoid present in fruits and vegetables, and as a supplement has an excellent safety profile and bioavailability^12, 13^. Quercetin has anti-inflammatory and antitumor activity, is an antioxidant, and has been reported to decrease the risk of chronic health conditions including cardiovascular and neurodegenerative diseases, diabetes, and tumorigenesis^14–16^.

CCl_4_ is a laboratory reagent characterized by toxicity causing liver damage and liver fibrosis and is extensively used in liver-related studies^17^. Bile duct ligature (BDL) impairs bile formation and bile flow, leading to cholestatic liver injury that progresses to hepatic fibrosis and cirrhosis^7^. Animal models of liver cirrhosis are widely used to study the mechanisms underlying liver fibrosis and the effect of various drugs on its progression^18–21^. This study investigated atypical signaling pathways in BDL and CCL_4_ mouse models of hepatic fibrosis. The involvement of PI3K and SMADs in modulation of TGF-β1-induced liver fibrosis and the effect of quercetin were assessed.

## Results

### Neither quercetin nor surgery alone had detectable effects on normal liver tissue

**Figure 1:**
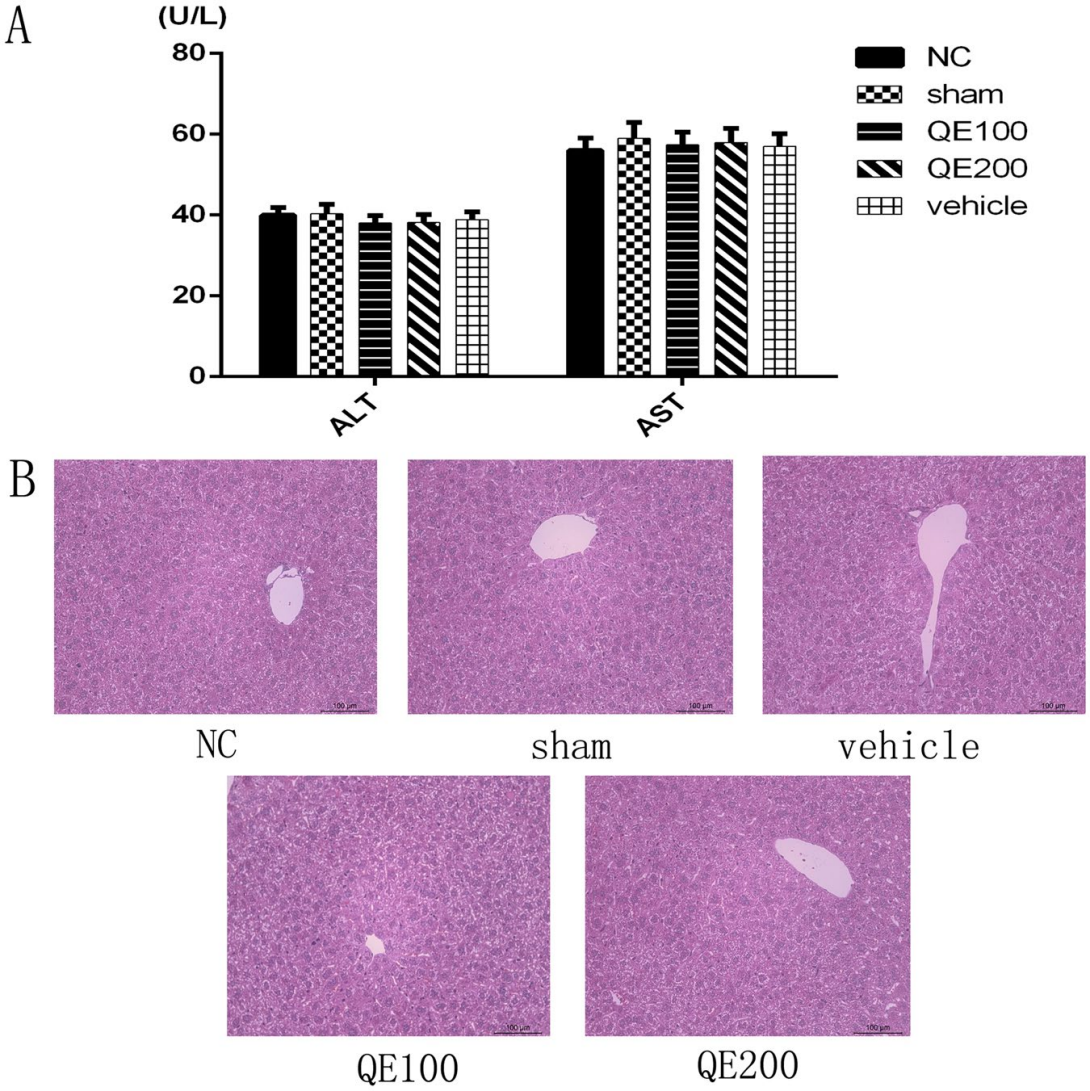
The effects of Quercetin and surgery on the liver function and pathology of healthy C57 mice. Notes: (**A**)The levels of serum ALT and AST in the five groups did not differ. Data are given as means ± SD(n = 5, P > 0.05). (**B**)Representative H&E stained sections of the liver (original magnification, ×200).

As shown in Fig. 1, there were no significant differences in the serum or liver transferase levels in the control, sham operation, vehicle and QE groups. No obvious pathological and morphological changes were visible in H&E stained tissues.

### Quercetin improved liver fibrosis induced by BDL or CCl_4_ in the C57 mouse models

**Figure 2:**
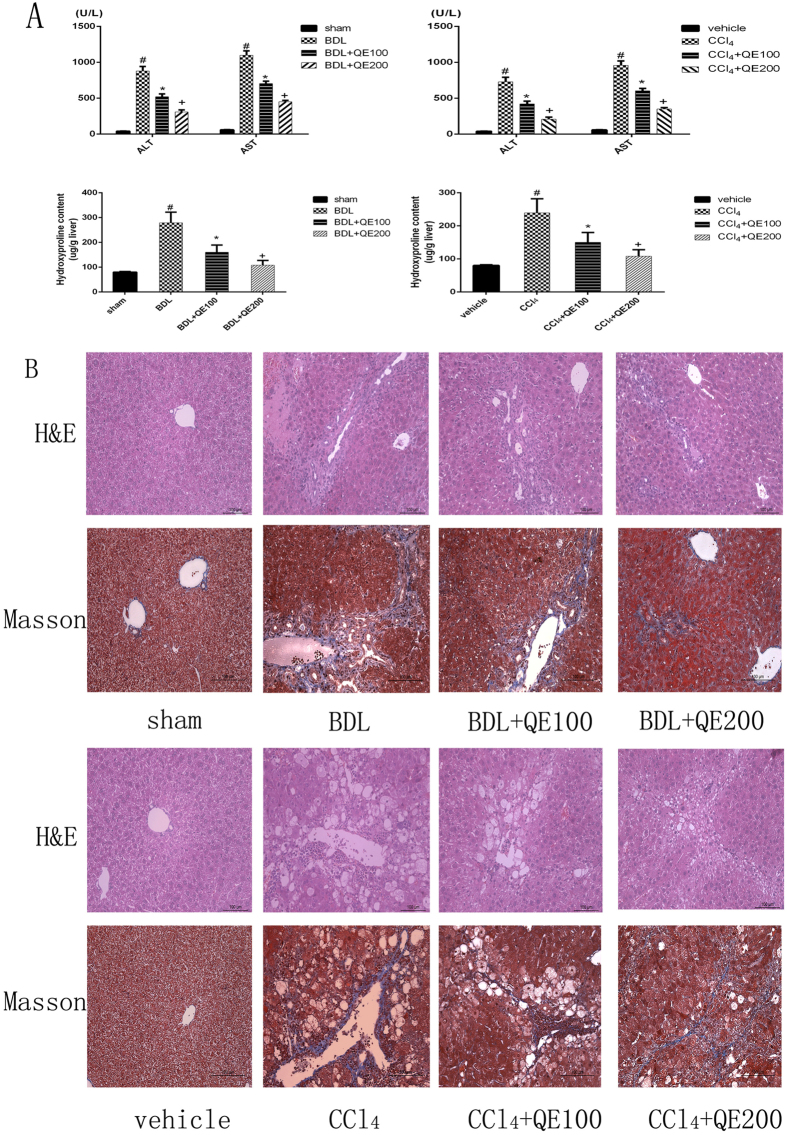
Effects of QE on liver function and pathology of C57 mice with liver fibrosis induced by BDL or CCl_4_. Notes: (**A**)The levels of serum ALT, AST and hydroxyproline content changed depending on the quercetin dose,100 mg/kg or 200 mg/kg. Data are given as means ± SD (n = 8, *P < 0.05 for sham or vehicle versus model group, ^#^P < 0.05 for BDL(CCl_4_) + QE100 versus model groups, and +P < 0.05 for BDL(CCl_4_) + QE200 versus model groups). (**B**) Quercetin ameliorated pathologic changes caused by fibrotic progression, showed by H&E and Masson’s trichrome (original magnification, ×200).

As shown in Fig. 2A, ASL and ALT were significantly increased in BDL- and CCl_4_- model mice compared with sham-operated or vehicle-treated mice. Quercetin treatment significantly decreased liver enzyme levels in a dose-dependent manner. Hydroxyproline, which increased in liver fibrosis, was significantly decreased by quercetin. Figure 2B shows the results of H&E and Masson staining of liver tissue from both BDL and CCl_4_ fibrosis models. In BDL-induced liver fibrosis, there were bundles of collagen fibers surrounding lobules with structural rearrangement of liver tissue. In CCl_4_-induced liver fibrosis, hyalinization of cytoplasm and vacuolated hepatocytes were present in addition to collagen fiber bundles. Analysis of Data (Table 1) confirmed that the average severity scores for liver fibrosis in quercetin-treated groups were markedly reduced in a dose-related fashion when compared with model groups. Quercetin markedly reduced the extent of tissue damage in BDL- and CCl_4_-model mice, confirming its protective effect.

**Table 1:** Degree of liver fibrosis in each group.

Group(CCl^4^)	N	Degree of liver fibrosis according to score					
		0	1	2	3	4	Mean
vehicle	8	8	0	0	0	0	0
CCl_4_ model	8	0	0	2	2	4	3.25^*^
CCl_4_ + QE100	8	0	1	3	3	1	2.5^#^
CCl_4_ + QE200	8	0	2	4	2	0	2^$^
**Group(BDL)**	**N**	**Degree of liver fibrosis according to score**					
		0	1	2	3	**4**	**Mean**
sham	8	8	0	0	0	0	0
BDL model	8	0	0	0	3	5	3.625^*^
BDL + QE100	8	0	0	4	3	1	2.625^#^
BDL + QE200	8	0	2	3	3	0	2.125^$^

Notes: *P < 0.05 for sham or vehicle versus model group, ^#^P < 0.05 for BDL(CCl_4_) + QE100 versus model groups, and ^$^P < 0.05 for BDL(CCl_4_) + QE200 versus model groups.

### Quercetin inhibited ECM formation and regulated MMP-9 and TIMP1 expression

**Figure 3:**
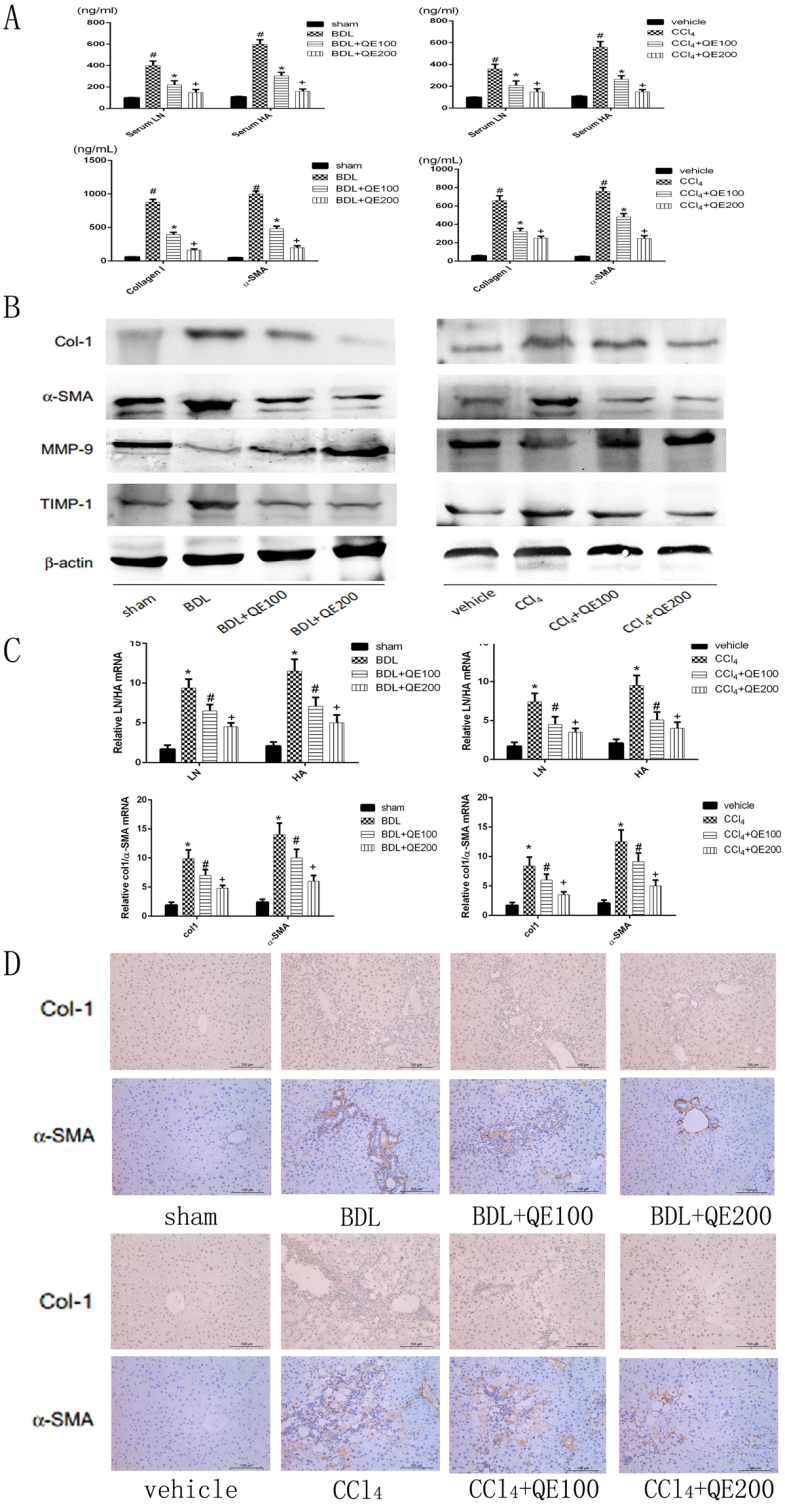
Effects of QE on the production of ECM in C57 mice with liver fibrosis induced by BDL or CCl_4_. Notes: (**A**) The levels of serum HA, LN and collagen I and α-SMA measured with ELISAs were reduced by quercetin treatment in mice at doses of 100 mg/kg and 200 mg/kg. Data are presented as means ± SD (n = 8, *P < 0.05 for sham or vehicle versus model group, ^#^P < 0.05 for BDL(CCl_4_) + QE100 versus model groups, and +P < 0.05 for BDL(CCl_4_) + QE200 versus model groups). (**B**) Quercetin changed the expression of Collagen I, α-SMA, MMP-9 and TIMP-1, which were detected by western blot. (**C**) The mRNA expression of LN, HA, Collagen I and α-SMA were measured by qRT-PCR. Data are presented as means ± SD (n = 8, *P < 0.05 for sham or vehicle versus model group, ^#^P < 0.05 for BDL(CCl_4_) + QE100 versus model groups, and +P < 0.05 for BDL(CCl_4_) + QE200 versus model groups). (**D**) Immunohistochemistry staining(×200) showing the expression of Collagen I and α-SMA in liver tissue, which were reduced by quercetin treatment.

As shown in Fig. 3A, serum HA, LN, Collagen I, and α-SMA were significantly increased in BDL- and CCl_4_-model mice compared with mice in the sham and vehicle groups, and both were decreased by quercetin in a dose-dependent manner. And the qRT-PCR results for LN and HA were accordance with serum results. Collagen I, α-SMA, MMPs and TIMPs also participated in the synthesis of ECM. qRT-PCR and western blots (Fig. 3B and C) show that Collagen I, α-SMA and TIMP1 expression were significantly increased in the fibrosis model groups, and that the increase was inhibited by quercetin. Similar results were obtained by immunohistochemical staining. The effect on MMP-9 expression was the opposite. MMP-9 expression was decreased in the fibrosis model groups, and was increased by quercetin treatment. Quercetin was thus found to inhibit ECM formation in both mouse models of liver fibrosis.

### Quercetin attenuated liver damage via the TGF-β1/Smads signaling pathway

**Figure 4:**
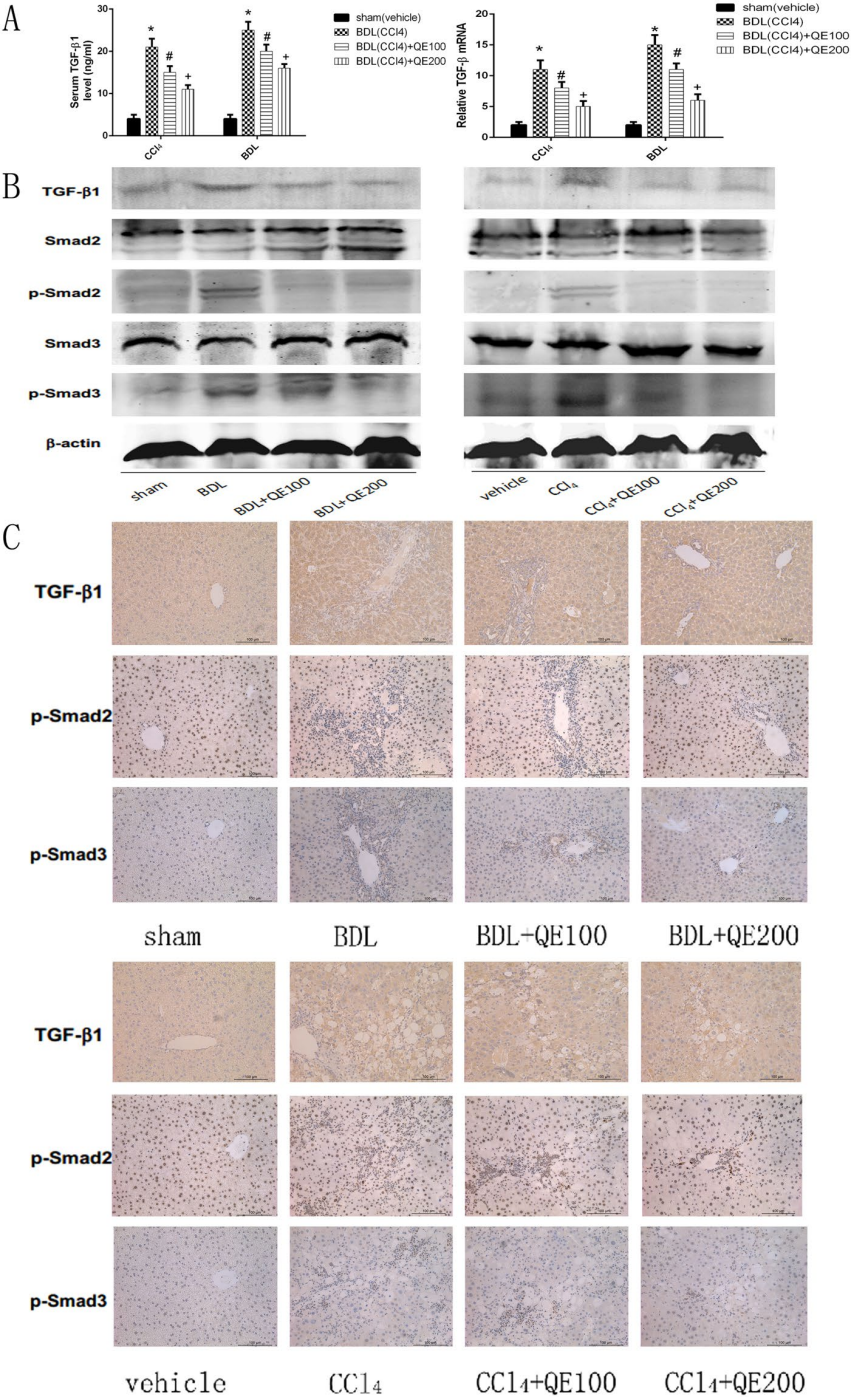
Effects of QE on TGF-β1/Smads signaling pathway in fibrotic liver. Notes: (**A**) The serum and mRNA levels of TGF-β1 was decreased by quercetin depending on its dose.Data are presented as means ± SD (n = 8, *P < 0.05 for sham or vehicle versus model group, ^#^P < 0.05 for BDL(CCl_4_) + QE100 versus model groups, and +P < 0.05 for BDL(CCl_4_) + QE200 versus model groups). (**B**) Quercetin effectively changed the expression of TGF-β1, p-Smad2 and p-Smad3, which were detected by western blot. (**C**) Immunohistochemistry staining(×200) showing the expression of TGF-β1, p-Smad2 and p-Smad3 in liver tissue, which were reduced by quercetin treatment.

TGF-β1 mRNA and protein expression in serum and tissues were up-regulated in both the BDL and CCl_4_ groups; quercetin down-regulated expression (Fig. 4A,B and C). Neither Smad2 nor Smad3 expression was affected (Fig. 4B), but p-Smad2 and p-Smad3 were both significantly increased in the fibrosis model groups. Quercetin treatment inhibited the in a dosage-dependent manner. The immunohistochemical staining, (Fig. 4C) western blot, and qRT-PCR results were consistent. Quercetin reduced TGF-β1 expression and inhibited the Smads signaling pathway in liver fibrosis.

### Quercetin attenuated liver damage via the PI3K/Akt signaling pathway

**Figure 5:**
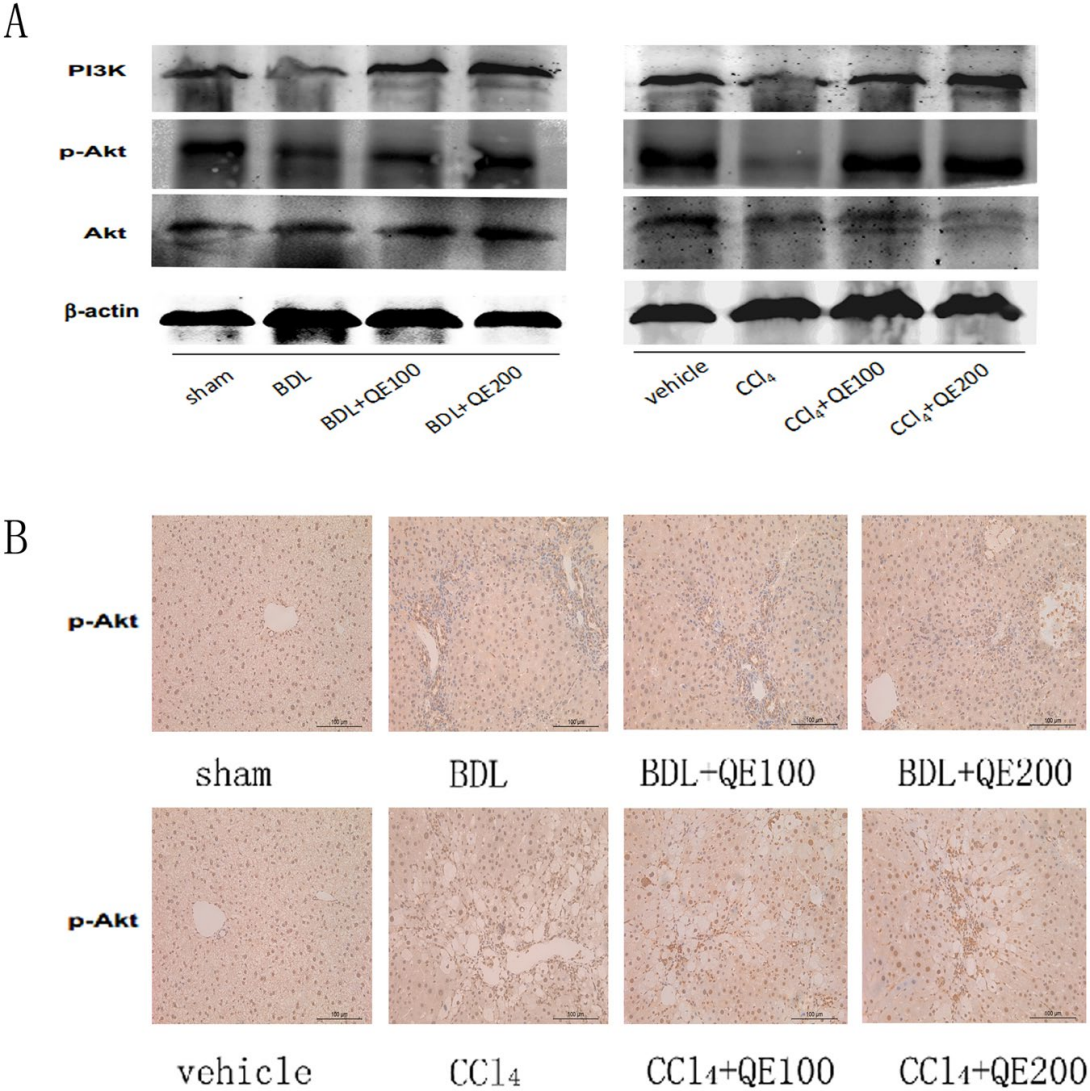
Effects of QE on the regulation of the PI3K/Akt signaling pathway in fibrotic liver. Notes: (**A**) Quercetin effectively changed the expression of p-PI3K and p-Akt, which were detected by western blot. (**B**) Immunohistochemistry staining(×200) showed the expression of p-Akt in liver tissues.

As shown in Fig. 5A, PI3K expression was suppressed in the fibrosis models and increased by quercetin treatment. Akt expression was not significantly changed in the fibrosis models, but p-Akt expression was increased by quercetin. Immunohistochemical staining revealed larger areas of p-Akt–positive tissue in quercetin-treated compared with untreated fibrosis model mice. Quercetin activated the PI3K/Akt signaling pathway in liver fibrosis.

### Quercetin could inhibit autophagy process in BDL- or CCl4- induced liver fibrosis

**Figure 6:**
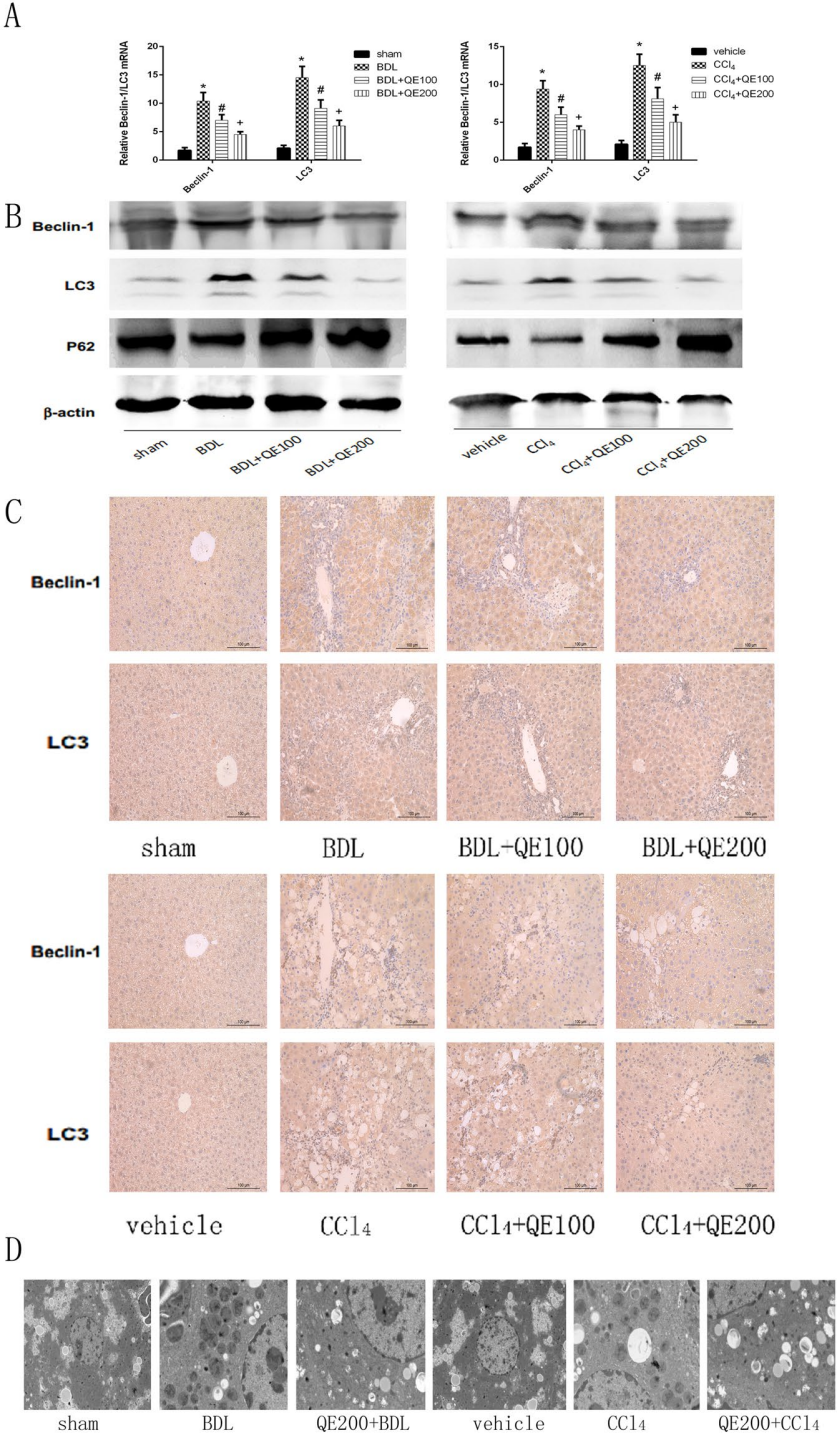
Effects of QE on autophagy in liver fibrosis. Notes: (**A**) The mRNA levels of Beclin-1 and LC3 were decreased by quercetin in a dose-depended manner. Data are presented as means ± SD (n = 8, *P < 0.05 for sham or vehicle versus model group, ^#^P < 0.05 for BDL(CCl_4_) + QE100 versus model groups, and ^+^P < 0.05 for BDL(CCl_4_) + QE200 versus model groups). (**B**) Quercetin effectively changed the expression of Beclin-1,LC3 and P62, which were detected by western blot. (**C**) Immunohistochemistry staining(×200) showed the expression of Beclin-1 and LC3 in liver tissues. (**D**) The formation of autophagosomes was shown by TEM(original magnificantion:1.2k for sham and vehicle;3.0k for model groups and treatment groups).

Changes in the expression of Beclin-1, LC3, and P62, which are markers of autophagosome formation, are shown in Fig. 6. Beclin-1 and LC3 mRNA and protein expression significantly increased in the BDL and CCl_4_ model mice, and quercetin effectively suppressed expression. P62 expression was decreased in the liver fibrosis model mice but was increased by quercetin treatment. (Fig. 6A) The immunohistochemical staining results were consistent with the qRT-PCR and western blotting shown in Fig. 6B. To make sure the influence of these markers on autophagy, we used electron microscopy to detected the formation of autophagosomes, whose results were shown in Fig. 6D. The number of autophagosomes in BDL and CCl4 groups were obviously increased, and in treatment groups, the agglutinated chromatin in mitochondria and autophagy corpuscles were not seldom seen. The results are consistent with quercetin inhibition of autophagy in fibrotic model mice.

## Discussion

Fibrosis is a frequent consequence of liver injury and can progress to cirrhosis and even hepatocarcinoma. The pathology of liver fibrosis is distinguished by activation of HSCs and excessive accumulation of ECM. HSC activation and proliferation are followed by replacement of hepatocytes by ECM, scar formation, and fibrosis^22^. The clinical benefits of quercetin have been described in diverse conditions affecting the heart, lungs, liver, skin, and bone, including renal^23, 24^ and pulmonary fibrosis^25^. Previous studies that evaluated the effects of quercetin on hepatic fibrosis did not include signaling pathways or autophagy^26–30^. This study investigated the antifibrotic effects of quercetin and the involvement of signaling pathways and autophagy in experimental mouse CCl_4_ and BDL hepatic fibrosis models.

Leakage of hepatic enzymes is a marker of hepatotoxicity. Serum ALT and AST were significantly increased in CCl_4_ and BDL model mice, but were reduced by quercetin. Hydroxyproline is a constituent of collagen and a marker of collagen deposition^25^. Liver hydroxyproline content was significantly increased in fibrosis model mice compared with controls, and was significantly decreased in the quercetin treatment groups. In line with those results H&E and MT staining indicated that in fibrosis model mice, the liver lobule structure was replaced by paraplastic connective tissues and extensive development of fibrosis. Deposition of abnormal amounts or excess collagen is characteristic of liver fibrosis, and the damage of liver tissues was decreased by quercetin. The results consistently demonstrated that the administration of quercetin effectively inhibited both CCl_4_ and BDL-induced hepatic fibrosis.

Fibrosis is a consequence of tissue repair and is closely associated with remodeling of the ECM. HA and LN levels are thought to reflect the degree of fibrosis, both were increased in fibrotic model mice, and were decreased by quercetin treatment. α-SMA and Collagen I were also over expressed in fibrosis model mice and were down-regulated by quercetin. MMPs and TIMPs are secreted enzymes involved in ECM degradation. In model mice, MMP9 expression was suppressed and TIMP1 expression was increased, those changes were reversed by quercetin treatment. Consequently, the ratio of MMP9:TIMP1 was increased by quercetin compared with untreated model mice. Quercetin thus promoted matrix degradation, most likely by suppressing the activation of HSCs.

TGF-β is involved in liver fibrosis,^31, 32^ promoting progression by both autocrine and paracrine mechanisms^33, 34^. Studies in adenovirus transfected or transgenic revealed that TGF-β contributed to HSC activation and fibrotic damage, and that blocking TGF-β signaling inhibited the fibrotic process^35, 36^. TGF-β1/Smads signaling is required for fibrosis^20^. As expected, TGF-β1, p-Smad2, and p-Smad3 were all up-regulated by liver injury, and all were suppressed by quercetin treatment. Smad2 and Smad3 expression did not significantly change. The expression of TGF-β1 in HSCs is regulated by the phosphorylation of Smad2 and Smad3, which occurs following TGF-β1 binding with its transmembrane receptor, TGF-β receptor I, and complexing with Smad4. The complex is transported into the nucleus where it binds to transcription factors for collagen and TIMPs^37, 38^. In this study, quercetin inhibited TGF-β signaling, which is in agreement with other reports^39^. The inhibitory effect of quercetin in fibrotic liver may be closely related to a decrease of TGF-β generation.

The phosphatidylinositol 3-kinase (PI3K)-protein kinase B (PKB/Akt) pathway modulates cell proliferation, survival, and motility, and glucose metabolism. Once, PI3K generates 3’-phosphoinositides [PI(3,4)P2 and PI(3,4,5)P3], which then recruit target proteins, such as Akt, to the plasma membrane. Akt, a Ser-Thr kinase, is an effector of PI3K, and initiates a kinase cascade that regulates cellular activities. The PI3K/Akt pathway is known to be involved in the progression of liver fibrosis,^40–42^ and expression of PI3K and p-Akt, the active form of Akt in hepatocytes, was detected in liver tissues in this study. The results were as expected, with expression inhibited in the fibrosis models and up-regulated by quercetin. Previous studies confirmed that the PI3K/Akt pathway could stimulate HSC proliferation, inhibit HSC apoptosis, and modulate the development and progression of liver fibrosis via its effect on ECM degradation^43, 44^.

**Figure 7:**
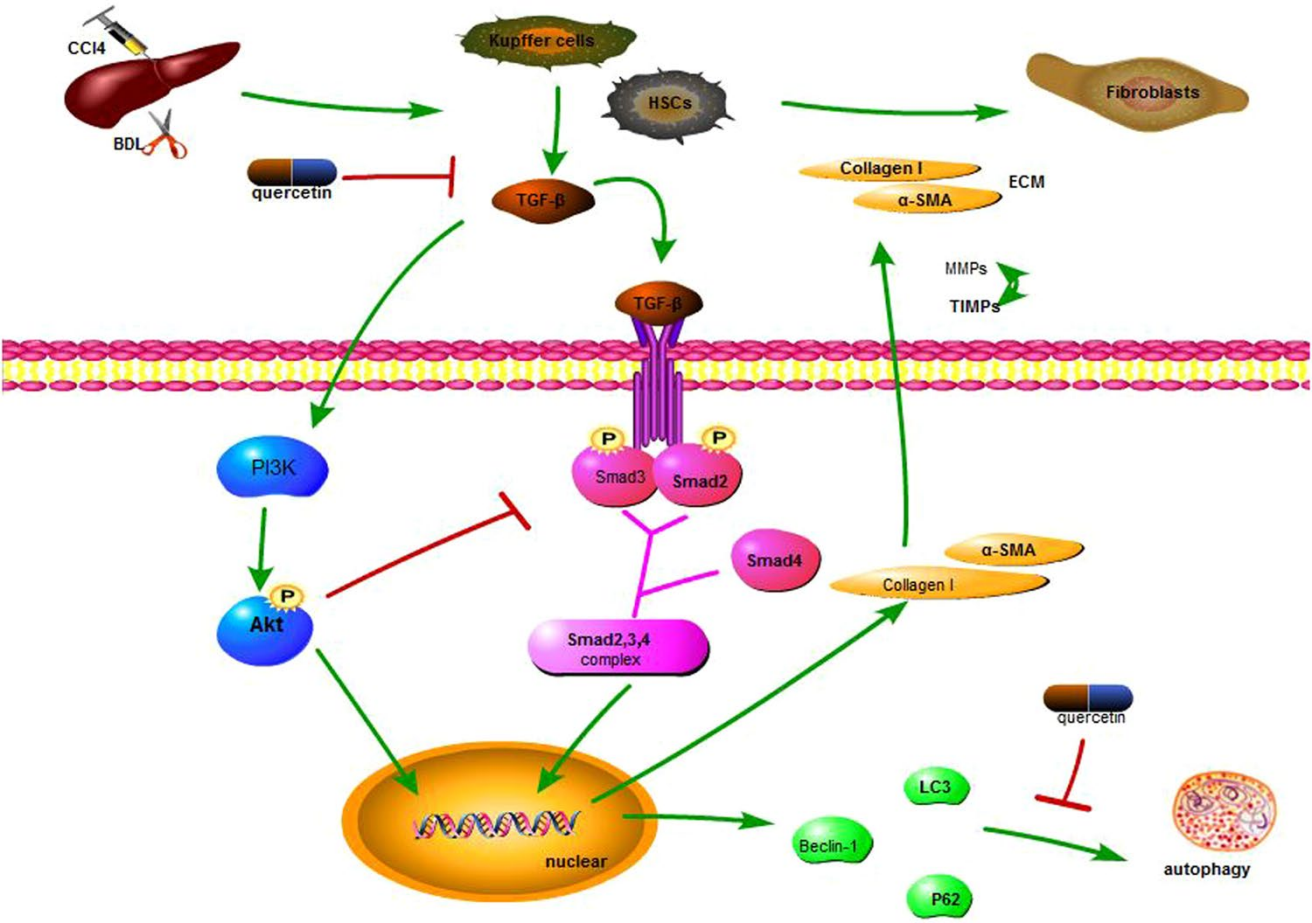
Mechanism of QE action. Notes: Quercetin inhibited the secrete of TGF-β1, which affected the TGF-β1/Smads signaling pathway and PI3K/Akt signaling pathway. When TGF-β1 bound with its trans-membrane receptors, Smad2 and Smad3 were activated and form a complex with Smad4.The complex then moved into the nuclear and regulate its downstream effectors. At the same time, quercetin activated the PI3K/Akt signaling pathway, which also took actions to modulate cellular actions. These two pathway had a complex cross talk in cytoplasma, and finally inhibited autophagy progression.

The PI3K/Akt and TGF-β1/Smads pathways have similar modes of action, and both were involved in our mouse fibrosis model(Fig. 7). When p-Smad3 signals were over expressed, Akt and PI3K were inhibited. When the p-Smad3 expression was reduced, phosphorylation of Akt was increased compared with mice in the vehicle and sham groups. TGF-β1 has been shown to activate PI3K, with phosphorylation of Akt, and subsequently activation of downstream effectors that affect cell growth and survival^45^. Akt has been shown to inhibit Smad3-mediated growth inhibition and apoptosis by binding to and sequestering Smad3 in the cytosol^46, 47^. Akt can also block the cytostatic activity of TGF-β by phosphorylation of Forkhead box O (FOXO), which prevents nuclear translocation and FOXO-Smad complex formation. The inhibition of p15^INK4B^ and p21^CIP1^ expression also led to loss of TGF-β function.^48^ Cross talk between the PI3K/Akt and TGF-β1/Smads pathways is complex, and may produce different effects depending on the cellular context and the biological processes involved^49, 50^. The significance of the crosstalk needs further study.

Autophagy is a key regulator of liver homeostasis and is responsible for the degradation of cell components by lysosomes. LC3 plays an important role in the formation of autophagosomes, the autophagy-related gene regulates autophagy, Beclin-1 (Atg6) is a marker of autophagy, and P62 is an autophagy adaptor protein^51^. Autophagy can be activated by TGF-β1 signaling^52^ and activation of the PI3K/Akt pathway in hepatocytes^53^. In these hepatic fibrosis models, increased expression of Beclin-1 and LC3 and a decreased expression of P62 are consistent with HSC activation by autophagy. Quercetin reduced Beclin-1 and LC3 expression and increased P62 expression, indicating that it slowed the progression of fibrosis by inhibition of autophagy.

Further, we plan to do more studies on the treatment of quercetin for liver diseases, such as hepatitis^54, 55^, inflammation associated hepatic diseases^56^, and hepatocellular carcinoma^57–59^. And we hope that quercetin can delay the progression of liver diseases, and cure them.

## Material and Methods

### Reagents

Quercetin was purchased from Sigma–Aldrich (St. Louis, MO, USA) and CCL4 from Signopharm (Shanghai, China). Aspartate aminotransferase (AST) and alanine aminotransferase (ALT) microplate test kits were from Jiancheng Bioengineering Institute (Nanjing, China). Collagen I, beclin-1, MMP2, TIMP1, α-SMA, PI3K, Akt, p-Akt, and p62 antibodies were purchased from Proteintech (Chicago, IL, USA). Anti-TGF-β1, Smad2, Smad3, p-Smad2, and p-Smad3 were from Abcam (Cambridge, MA, USA). Anti-LC3 was purchased from Cell Signaling Technologies (Beverly, MA, USA). The PCR kit was from Takara (Takara Biotechnology, Dalian, China).

### Animals

Male C57 mice weighing 23 ± 3 g were purchased at 8 weeks of age from Shanghai SLAC Laboratory Animal Co., Ltd. (Shanghai, China). The mice were raised in a standard environment with ad libitum food and water. Animal experiments were performed following National Institutes of Health Guidelines and were approved by the Animal Care and Use Committee of Shanghai Tongji University.

### Preliminary study

Twenty mice were randomly divided into four groups of five mice each. The control group received no treatment. The vehicle group received saline by gavage once daily for 2 weeks. The QE group were given 200 mg/kg QE by gavage once daily for 2 weeks, and the sham group mice were given a sham operation. QE was dissolved in saline, mice were sacrificed after 2 weeks and serum and liver tissue were collected for ALT and AST assays and pathological evaluation.

### Fibrosis induction

In the CCl_4_ fibrosis model, mice were injected intraperitoneally with 1.0 mL/kg 10% CCl_4_ ν/ν in olive oil for 8 weeks. Quercetin 100 mg/kg or 200 mg/kg in saline was given at the same time by gavage. Mice were randomly divided into four groups of eight mice each. The vehicle group: mice received saline by gavage. The CCl_4_ group received daily injections of CCl_4_. The CCl_4_ + quercetin (100) group received CCl_4_ and 100 mg/kg quercetin daily, and the CCl_4_ + quercetin (200) group received CCl_4_ and 200 mg/kg quercetin daily.

In the BDL model, mice were fasted for about 24 hand then were anesthetized with 1.25% sodium pentobarbital (Nembutal, St. Louis, MO, USA) and placed on a sterile, heated table. A midline laparotomy was performed; the bile duct was exposed with a wet swab, ligated with two surgical knots, and cut between knots. The abdominal cavity was then closed. After BDL, the mice were given quercetin orally for 2 weeks. BDL mice were randomly divided into four groups of eight mice each. These were a sham group, a BDL only group, a BDL + quercetin (100) group, and a BDL+ quercetin (200) group. Liver tissue and blood samples were collected from all animals for histological evaluation and molecular assays.

### Serum assays

Serum was separated from blood samples by centrifugation at 4600 *g* for 10 minutes, and was stored at –80 °C until use. ALT and AST were assayed with an automated chemical analyzer (Olympus AU1000, Olympus, Tokyo, Japan). Hydroxyproline, HA, LN, collagen I, and *a*-SMA were assayed with commercial kits following the manufacturers’ instructions.

### Histopathology and Fibrosis Score

Liver tissue samples were fixed in 4% paraformaldehyde and embedded in paraffin after dehydration in an ethyl alcohol series. Serial sections were cut at 5 μm and stained with hematoxylin and eosin (H&E) and Masson’s trichrome (MT), and evaluated by light microscopy.

Following is the criteria of liver fibrosis severity score(Table 2).

**Table 2:** Fibrosis Score.

No fibrosis	Normal	o
collagen fibers present that extends from portal triad or central vein to peripheral region	fibrosis present	1
mild collagen fiber present with extension but without compartment formation	mild fibrosis	2
moderate collagen fibers present with some pseudo lobe formation	moderate fibrosis	3
many collagen fibers present with thickening of the partial compartments and frequent pseudo lobe formation	severe fibrosis	4

### Western blotting

Total protein was extracted following standard procedures, and samples were stored at –20 °C until separated on sodium dodecyl sulfate polyacrylamide gels and transferred onto nitrocellulose (NC) or polyvinylidene difluoride (PVDF) membranes. After blocking with 5% nonfat milk for more than 1 h, membranes were incubated with primary antibodies at 4 °C overnight. The membranes were then washed in phosphate buffered saline(PBS) with Tween 20 (PBST; 0.1 mL Tween in 1 L PBS) three times for 10 min each. The membranes were incubated with secondary goat anti-mouse or anti-rabbit antibodies at room temperature for 1 h. After washing in PBST for 30 min, the membranes were scanned using an Odyssey two-color infrared laser imaging system (LI-COR Biosciences, Lincoln, NE, USA).

### Immunohistochemistry Staining

Slides with 5 μm serial sections were dewaxed, and rehydrated. They were then immersed in citrate buffer (pH 6.0) heated to 60 °C for 1 hour for antigen retrieval, covered with 3% hydrogen peroxide solution for 20 minutes at 37 °C to block endogenous peroxidases, and washed in PBS. Sections were immersed in 5% bovine serum albumin at 37 °C for 20 min and then incubated overnight at 4 °C with Beclin-1, LC3, TGF-β1, collage I, α-SMA, or p-Akt primary antibodies at dilutions of 1:500 each. The next day, sections were incubated with secondary antibody for 30 min followed by color development using a diaminobenzidine kit. The stained sections were observed by light microscopy. The integrated optical densities of different indices were calculated using Image-Pro Plus software 6.0 (Media Cybernetics, Silver Spring, MD, USA).

### Electron Microscopy

After prefixed with 3% glutaraldehyde buffered with 0.2 mmol/L cacodylate for 4 hours,the liver tissues were postfixed in 1% osmium tetroxide for 1 hour. And autophagosomes were observed by electron microscopy (JEM-1230; JEOL, Tokyo, Japan), and images were acquired.

### Reverse-transcription(RT)-PCR and quantitative real-time -PCR

Total RNA was extracted from liver tissue by TRIzol (Tiangen Biotech [Beijing] Co, Ltd, Beijing, China) and then reverse transcribed into cDNA the kit manufacturer’s instructions. The expression of target genes expression was assayed by SYBR Green qRT-PCR using a 7900HT fast real-time PCR system (Applied Biosystems, Foster City, CA, USA). The primer sequences used in this study are shown in Table 3.

**Table 3:** Nucleotide sequences of primers used for qRT-PCR.

Gene		Primer sequence(5′-3′)
TGF-β1	Forward	CCACCTGCAAGACCATCGAC
	Reverse	CTGGCGAGCCTTAGTTTGGAC
α-SMA	Forward	CCCAGACATCAGGGAGTAATGG
	Reverse	TCTATCGGATACTTCAGCGTCA
Beclin-1	Forward	ATGGAGGGGTCTAAGGCGTC
	Reverse	TGGGCTGTGGTAAGTAATGGA
LC3	Forward	GACCGCTGTAAGGAGGTGC
	Reverse	AGAAGCCGAAGGTTTCTTGGG
Collagen I	Forward	CAATGGCACGGCTGTGTGCG
	Reverse	AGCACTCGCCCTCCCGTCTT
TIMP1	Forward	CGAGACCACCTTATACCAGCG
	Reverse	ATGACTGGGGTGTAGGCGTA
MMP2	Forward	GGACAAGTGGTCCGCGTAAA
	Reverse	CCGACCGTTGAACAGGAAGG
laminin	Forward	CCCCGTCCTTGGATGTACCT
	Reverse	CAGTAGGGTTGAGGGCTATGC
hyaluronic acid	Forward	AGCAAGGATGGAATGACAACAG
	Reverse	TCAGTCTCATAGGGCCGGTAT
β-actin	Forward	GGCTGTATTCCCCTCCATCG
	Reverse	CCAGTTGGTAACAATGCCATGT

### Statistical analysis

Statistical analysis was performed using SPSS 20.0 software (IBM Corporation, Armonk, NY, USA). All data were expressed as means ± standard deviation (SD). A P-value < 0.05 was considered statistically significant. Figures were drawn using GraphPad Prism, v 6.0.
